# Raw Milk Consumption among Patients with Non–Outbreak-related Enteric Infections, Minnesota, USA, 2001–2010

**DOI:** 10.3201/eid2001.120920

**Published:** 2014-01

**Authors:** Trisha J. Robinson, Joni M. Scheftel, Kirk E. Smith

**Affiliations:** Minnesota Department of Health, St. Paul, Minnesota, USA

**Keywords:** foodborne diseases, foodborne illnesses, milk, dairy products, enteric pathogens, Minnesota, raw milk consumption, enteric infections, hemolytic uremic syndrome, bacteria, bacterial enteric pathogens, protozoal enteric pathogens, campylobacter, cryptosporidium, Shiga toxin–producing Escherichia coli, STEC, STEC O157, STEC non-O157 serogroups, salmonella, Escherichia coli

## Abstract

Raw milk has frequently been identified as the source of foodborne illness outbreaks; however, the number of illnesses ascertained as part of documented outbreaks likely represents a small proportion of the actual number of illnesses associated with this food product. Analysis of routine surveillance data involving illnesses caused by enteric pathogens that were reportable in Minnesota during 2001–2010 revealed that 3.7% of patients with sporadic, domestically acquired enteric infections had reported raw milk consumption during their exposure period. Children were disproportionately affected, and 76% of those <5 years of age were served raw milk from their own or a relative’s farm. Severe illness was noted, including hemolytic uremic syndrome among 21% of *Escherichia coli* O157–infected patients reporting raw milk consumption, and 1 death was reported. Raw milk consumers, potential consumers, and policy makers who might consider relaxing regulations regarding raw milk sales should be educated regarding illnesses associated with raw milk consumption.

## Abstract 2

The risk for illness associated with raw milk consumption is far greater than previously realized.

Raw milk is well-established as a vehicle for numerous infectious diseases (*1**–**8*) and has frequently been identified as the source of outbreaks of foodborne illness (*9**,**10*). From 1998 through 2011, a total of 148 outbreaks were documented in the United States associated with the consumption of raw milk products, resulting in 2,384 illnesses, 284 hospitalizations, and 2 deaths (*11*). A recent report concluded that the incidence of reported outbreaks associated with raw dairy products was ≈150 times greater, per unit of product consumed, than the incidence involving pasteurized dairy products (*12*).

Although pasteurization has been available for use in the United States for over a century, a small proportion of the population continues to consume raw milk. In a 2006–2007 population survey comprising 10 states in the Foodborne Diseases Active Surveillance Network, 3.0% of respondents reported consuming raw milk during the previous 7 days (*13*). Farm families have traditionally been the primary consumers of raw milk, but evidence in recent years suggests that the population of raw milk consumers may be changing (*14*). Raw milk advocates tout raw milk for its purported health benefits and better taste, and many persons view raw milk consumption as an opportunity to support local dairies (*15**,**16*). Some raw milk advocates fail to acknowledge the elevated health risk associated with raw milk consumption and minimize the significance of reported outbreaks. In doing so, these advocates convey a false sense of the safety of raw milk to those who are considering consuming this product, and this sense of safety discourages a balanced assessment of the potential risks and benefits involved.

Although outbreaks associated with raw milk occur frequently and receive much media attention, the number of reported cases determined to be outbreak-related likely represents a small proportion of the actual number of illnesses associated with this product. Two lines of evidence support this assumption. First, among reported illnesses caused by enteric pathogens that are laboratory-confirmed, non–outbreak-related (i.e., sporadic) cases far outnumber those associated with recognized outbreaks (*17*). Second, for each reported laboratory-confirmed illness caused by a bacterial or protozoal enteric pathogen, an estimated 26–100 additional illnesses likely occur, depending on the pathogen (*18*). Therefore, any estimates of the number of illnesses associated with raw milk consumption should include an evaluation of sporadic cases, including multipliers to account for underdiagnosis and underreporting. However, little information is available on the number of sporadic cases of illness associated with raw milk consumption.

Minnesota is among the 30 US states that permit raw milk to be sold in some capacity (*19*), allowing for raw milk to be occasionally secured or purchased for personal use at the farm or place where the milk is produced (*20*). To better estimate the true number of human enteric pathogen infections associated with consumption of raw milk, we characterized sporadic enteric illnesses that occurred among patients in Minnesota who reported raw milk consumption during 2001–2010. Our primary objective was to provide better data on the true number of sporadically occurring disease cases associated with raw milk consumption.

## Methods

Patients were identified through routine disease surveillance conducted at the Minnesota Department of Health (MDH). Infections caused by *Campylobacter*, *Cryptosporidium*, Shiga toxin–producing *Escherichia coli* (STEC, including O157 and non-O157 serogroups), and *Salmonella* are reportable to MDH by state rule, and active, population-based surveillance is conducted (*21*). All Minnesota residents with a laboratory-confirmed *Campylobacter*, *Cryptosporidium*, STEC O157, non-O157 STEC, or *Salmonella* infection are routinely interviewed by MDH staff using a disease-specific standard questionnaire about symptoms and food, water, animals, and other possible sources of infection during their exposure period. Each questionnaire contains a question about raw milk consumption, including where the raw milk was obtained and when it was consumed. Exposure period is defined as 7 days before illness onset for *Campylobacter*, STEC O157, non-O157 STEC, and *Salmonella* infections and 14 days for *Cryptosporidium* infection.

A case-patient was defined as a Minnesota resident who had a domestically acquired, laboratory-confirmed *Campylobacter*, *Cryptosporidium*, STEC O157, non-O157 STEC, or *Salmonella* infection and a specimen collection date during 2001–2010. Persons were excluded if they refused an interview or were unable to be reached for interview, were part of an outbreak, or traveled internationally during the exposure period. Patients infected with *Campylobacter upsaliensis*, *Cryptosporidium hominis*, and *Salmonella* *enterica* serotype Typhi were also excluded because these specific species or serotypes have not been documented to be associated with raw milk consumption or other cattle exposures. Patients infected with an unknown species or serotype were eligible for inclusion. The total number of case-patients reporting raw milk consumption was calculated, and we examined the demographic features, severity of illness, and raw milk sources among the case-patients. Estimates of the total number of illnesses that could be attributed to raw milk consumption were calculated by using published pathogen-specific multipliers that account for underdiagnosis (*18*).

Descriptive and univariate analyses were performed by using SAS 9.2 software (SAS Institute, Cary, NC, USA). Case-patients who refused to answer a question or responded don’t know or not sure were excluded from relevant analyses. Statistical significance was accepted at p<0.05.

## Results

During 2001–2010, a total of 20,034 *Campylobacter*, *Cryptosporidium*, STEC O157, non-O157 STEC, and *Salmonella* infections were reported to MDH. Among these cases, 6,695 were excluded for the following reasons: the patient reported international travel (2,648 cases) or refused or was unable to be reached for interview (1,530 cases); the patient was linked to a recognized outbreak (1,244 cases); or the infection was caused by a species or serotype not historically associated with raw milk or other cattle exposures (273 cases).

Of the excluded outbreak cases, 21 occurred during 5 recognized outbreaks associated with raw milk consumption in Minnesota during 2001–2010. These 5 outbreaks resulted in 7 hospitalizations and 1 case of hemolytic uremic syndrome (HUS). One outbreak of *Campylobacter jejuni* infections in 2001 was associated with raw milk consumption at a farm where a ministry group was staying. Two outbreaks of *C. jejuni* infections occurred in 2008: one was associated with raw milk consumption at a family reunion and the other with raw milk purchased from a local dairy farm. In 2010, an outbreak of STEC O157 infections and an outbreak of *C. jejuni* and *Cryptosporidium parvum* infections were associated with consumption of raw milk from the same dairy farm; both outbreaks included several cases associated with milk the consumers had picked up at illegal drop-off sites.

**Figure 1:**
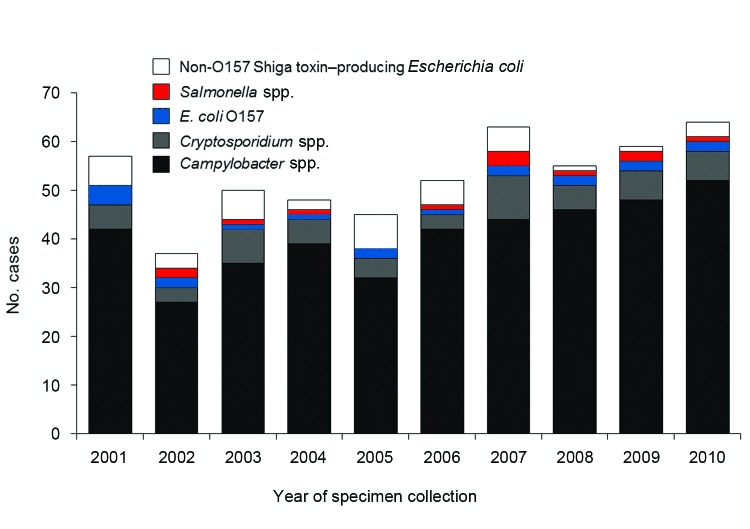
Number of cases of domestically acquired, sporadic enteric infections for which the patient reported raw milk consumption, by year and pathogen (n = 530), Minnesota, 2001–2010.

After exclusions, a total of 14,339 cases remained for analysis, including 6,747 *Campylobacter* spp., 1,742 *Cryptosporidium* spp., 1,069 STEC O157, 354 non-O157 STEC, and 4,427 *Salmonella* spp. cases. Among the 14,339 patients, 530 (3.7%) reported consumption of fluid raw milk during their exposure period (Table 1). The median annual number of case-patients reporting raw milk consumption was 53.5 (range 37–64), but this number generally increased over time (Figure 1). Among the 273 persons who were excluded from study because of infection with a species or serotype not historically associated with raw milk or other cattle exposures, only 2 (0.7%) reported raw milk consumption; this was significantly lower than the 3.7% of cases included in the analysis (p = 0.01).

**Table 1:** Demographic characteristics of patients with domestically acquired, sporadic enteric infections, Minnesota, 2001–2010

Pathogen	Patients reporting raw milk consumption				Patients denying raw milk consumption		
	No. patients	Median age, y (range)	Male, %		No. patients	Median age, y (range)	Male, %
*Campylobacter* spp.	407	18 (<1–92)*	63.9*		6,340	33 (<1–96)	56.9
*Cryptosporidium* spp.	53	8 (1–74)*	52.8		1,689	21 (<1–101)	45.6
*Escherichia coli* O157	19	5 (<1–63)*	63.2		1,050	16 (<1–92)	46.3
Non-O157 Shiga toxin–producing *E. coli*	12	4 (1–61)	66.7		342	18 (<1–88)	46.3
*Salmonella* spp.	39	16 (1–78)*	59.0		4,388	28 (<1–98)	46.6
Total	530	17 (<1–92)*	62.6*		13,809	29 (<1–101)	51.3

*Significantly different (p<0.05) from persons who denied raw milk consumption.

Persons with *Campylobacter* infection had the highest percentage of reported raw milk consumption (6.0%), and *Campylobacter* spp. accounted for 407 (77%) of the 530 cases with reported raw milk consumption (Table 1, Figure 1). Among case-patients infected with other pathogens, the percentage, by pathogen, reporting raw milk consumption included: non-O157 STEC, 3.4%; *Cryptosporidium* spp., 3.0%; STEC O157; 1.8%, and *Salmonella* spp., 0.9%. The following data were available regarding the speciation of pathogens from case-patients: 378 *Campylobacter* isolates (*C*. *jejuni*, 96.8%; *C. coli*, 2.6%; and *C. lari*, 0.5%); 23 *Cryptosporidium* *parvum* specimens; 36 *Salmonella* isolates (16 serotypes, most frequently *S*. *enterica* serotype Typhimurium, 10 isolates; *S*. *enterica* serotype Montevideo, 6 isolates; and *S*. *enterica* serotype Newport, 5 isolates).

Twelve patients were co-infected with *Campylobacter* spp. and 1 other enteric pathogen: 9 with *Cryptosporidium* spp., 1 with STEC O157, 1 with non-O157 STEC, and 1 with *Salmonella* spp. In addition, 1 patient who was reported to have consumed raw milk was infected with different pathogens at different times during the study period: STEC O157 (including HUS) at 1 year of age and *Salmonella* spp. 1 year later. 

**Figure 2:**
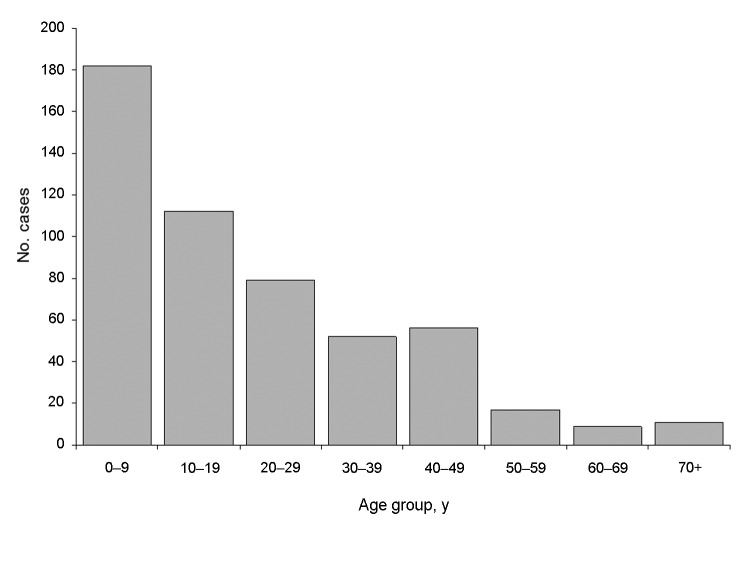
Age distribution among patients with domestically acquired, sporadic enteric infections who reported consumption of raw milk during their exposure periods (n = 518), Minnesota, 2001–2010.

Male case-patients comprised 62.6% of study participants reporting raw milk consumption (Table 1). Case-patients reporting raw milk consumption were more likely than the average Minnesotan to be white (96.5% vs. 85.3%; p<0.001), and 96.8% were non-Hispanic. Overall, the median age of case-patients reporting raw milk consumption was 17 years (range 9 months to 92 years); 25% were <5 years of age, 38% were <10 years of age, and 59% were <20 years of age (Figure 2). Among patients with STEC O157 infections, those reporting raw milk consumption had a median age of 5 years (range 11 months to 63 years), compared with a median age of 16 years (range 5 months to 92 years) among those who did not report raw milk consumption (p = 0.02). Likewise, patients with non-O157 STEC infections who reported raw milk consumption had a median age of 4 (range 1–63) years. Patients with *Cryptosporidium*, *Salmonella*, or *Campylobacter* infection who reported raw milk consumption had median ages of 9, 16, and 18 years, respectively. Illnesses occurring among patients reporting raw milk consumption were disproportionately distributed throughout the year; 35% (186/530) of specimen collection dates occurred during the months of June, July, or August (p<0.001).

Seventy (13%) case-patients reporting raw milk consumption required hospitalization for a median of 3 (range 2–27) days for their illness. HUS occurred among 4 (21%) of the 19 patients with STEC O157 infection, including 2 of 4 children <3 years of age. One (8%) of the 12 patients with non-O157 STEC infection developed HUS. An 11-month-old infant with STEC O157 infection died.

**Figure 3:**
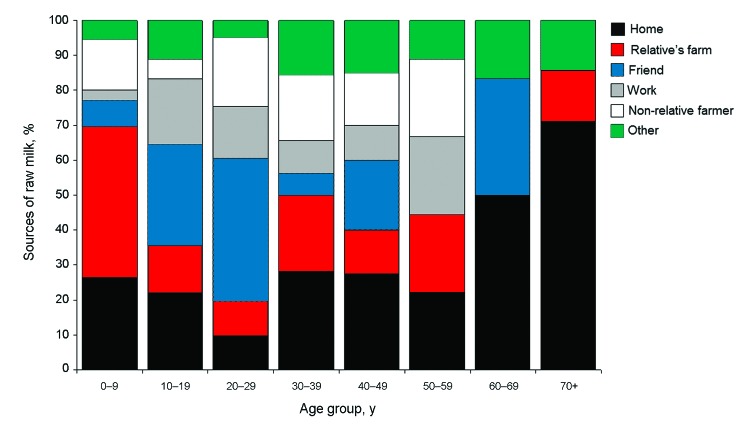
Distribution of reported raw milk sources, by patient age, among patients with domestically acquired, sporadic enteric infections who reported consumption of raw milk during their exposure periods (n = 377), Minnesota, 2001–2010.

Of the 530 case-patients who consumed raw milk, 377 (71%) provided information on the source of their milk, and almost half either obtained it from their own dairy farm (91 consumers, 24%) or from a relative’s dairy farm (90 consumers, 24%). Other reported sources included friends (73 consumers, 19%); nonrelative farmers, including direct farm sales (50 consumers, 13%); the workplace or a relative’s workplace (39 consumers, 10%); neighbors (16 consumers, 4%); drop-off sites (7 consumers, 2%); or other sources, including at daycare or school (11 consumers, 3%). Those who reported consuming raw milk from their own dairy farm or from a relative’s dairy farm were significantly younger than those who reported obtaining raw milk from a nonfamily source (median age 9 years vs. 19 years; p<0.001). Among children <5 years of age, 76% were reported to have consumed raw milk from their own dairy farm or a relative’s dairy farm; this proportion declined with age among pediatric patients, and a steep drop to 9% occurred among those 17–20 years of age, who more frequently reported obtaining raw milk from friends or at work (Figure 3). Among 464 case-patients with known information, 232 (50%) also reported contact with cattle or their environment during the exposure period; 68% of these exposures occurred in persons living or working on a farm or visiting a family member’s farm.

Pathogen-specific underdiagnosis multipliers (*18*) were applied to data regarding the 530 cases associated with reported domestic raw milk consumption and resulted in an estimate that 20,502 Minnesotans became ill with sporadic *Campylobacter*, *Cryptosporidium*, STEC O157, non-O157 STEC, or *Salmonella* infection during 2001–2010 after drinking raw milk (Table 2). We applied the percentage of Minnesotans in the Foodborne Diseases Active Surveillance Network population survey who reported consuming raw milk (2.3%) (*13*) to the 2006 state population (5,167,101) and determined that an estimated 118,843 Minnesotans consume raw milk during a given week. If these projected illness data are applied to the projected number of raw milk consumers and the percentage of Minnesotans who consume raw milk was consistently 2.3% during the study period, then an estimated 17.3% of raw milk consumers in Minnesota may have acquired an illness caused by 1 of these enteric pathogens during the 10-year study period.

**Table 2:** Number of sporadic illnesses resulting from raw milk consumption, as estimated by using underdiagnosis multipliers, Minnesota, 2001–2010

Pathogen	No. laboratory-confirmed cases	Multiplier to account for underdiagnosis (*18*)	Estimated no. actual cases
*Campylobacter* spp.	407	30.3	12,332
*Cryptosporidium* spp.	53	98.6	5,226
*Escherichia coli* O157	19	26.1	496
Non-O157 Shiga toxin–producing *E. coli*	12	106.8	1,282
*Salmonella* spp.	39	29.9	1,166
Total	530	–	20,502

## Discussion

Our study quantifies the number of non–outbreak-related enteric illnesses that could be associated with raw milk consumption. The results indicate that the number of sporadic raw milk–associated illnesses is likely substantial, greatly exceeding the number of cases linked to recognized raw milk–associated outbreaks. Furthermore, the number of cases associated with reported raw milk consumption appears to be increasing, just as the movement to relax regulation of raw milk sales appears to be gaining momentum in many states.

We found that young children were disproportionately affected, and the source of the raw milk they consumed was often their own dairy farm or a relative’s farm. This reinforces the risks associated with raw milk consumption for all young children, including those who live on or visit farms. Half of the STEC O157–related and non-O157 STEC–related illnesses occurred among children <5 years of age; this finding is of particular concern because of the increased risk for HUS among this age group (*22*). In our study, HUS developed in 21% of patients with STEC O157 infections who reported drinking raw milk, and 1 of these patients died. Because of the potential for severe health consequences associated with the consumption of raw milk, the American Academy of Pediatrics and several other professional organizations have adopted a position statement advising that children should only consume milk products that are pasteurized (*23*).

Our study suggests that farm family members, particularly young children, who consume raw milk are susceptible to illness from it. Results of previous studies have shown that 30%–50% of dairy producers surveyed were unaware that their raw bulk tank milk could contain disease-causing microorganisms (*24**,**25*). Therefore, dairy farm families, in particular, should be educated regarding this issue. The differences in disease risk by age group may be a result of acquired immunity among older adolescents and adults who were exposed to pathogens during farm exposures in childhood (*26*).

Most raw milk consumers included in our study were infected with *Campylobacter* spp., and these bacteria have repeatedly been associated with raw milk consumption, both in outbreak investigations and in case–control studies (*27**,**28*). Because of the complexities involved with molecular subtyping for *Campylobacter* spp. (*29*) and because of the high proportion of patients who do not report a source for their raw milk, it is likely that some of the apparently sporadic infections associated with raw milk consumption are actually illnesses related to unrecognized outbreaks.

This study provides evidence that raw milk is also a vehicle for *Cryptosporidium* spp., the second most common pathogen identified among raw milk drinkers. Cryptosporidiosis is a common infection in cattle, and contact with infected animals or their environment is frequently associated with human cryptosporidiosis (*30**,**31*). Although few cryptosporidiosis outbreaks associated with raw milk consumption have been reported (*6*), findings of other studies include an association between raw milk consumption and cryptosporidiosis (*32**,**33*).

We noted an increase in enteric disease cases associated with reported raw milk consumption during the summer months (June–August) compared with other seasons. This trend is in sync with the general seasonality of the enteric pathogens included in this study, and it is also consistent with data from recent studies on the seasonal incidence of *Salmonella* spp. sampled from bulk tank milk, dairy cows, and farm environments (*34*) and the seasonal trend of fecal shedding of STEC O157 by dairy cattle (*35*).

This study had several potential limitations. First, the cases were sporadic, so illness among the raw milk consumers could not be definitively linked to their raw milk consumption without overestimating the number of illnesses associated with raw milk consumption. For example, illness among some patients could have been associated with direct contact with cattle rather than raw milk consumption. However, 50% of patients did not report cattle contact, and the high-risk nature of raw milk consumption makes it the most likely source for most of the patients who did report concomitant cattle contact. This conclusion is supported by the close demographic and illness similarities among patients with reported raw milk consumption in our study and the outbreak cases reported by Langer et al. (*12*). It is also supported by the very low (0.7%) background level of raw milk consumption among patients with pathogens of a species or serotype not historically associated with raw milk consumption or other cattle exposures. 

The second potential limitation is that not all specimens of the pathogens of interest were identified to species or serotype; this was most pertinent for *Cryptosporidium* spp. Therefore, it is possible that some patients were misclassified as eligible, thus underestimating the percentage of raw milk drinkers. The third potential limitation is that refusals to participate in an interview or to answer questions about raw milk could be biased toward raw milk consumers. In contrast to the first limitation, this could potentially have resulted in an underestimation of the number of illnesses associated with raw milk consumption. The fourth potential limitation is that the data available to us for this study did not permit estimation of a true absolute risk for illness associated with raw milk consumption. The fifth and last potential limitation is that the pathogen-specific underdiagnosis multipliers have confidence intervals that were not considered here; therefore, estimates of raw milk–associated illnesses could be substantially higher or lower than we have reported (*18*).

Sporadic cases of illness associated with raw milk consumption far outnumber cases associated with recognized outbreaks. During the study period, the number of patients with sporadic laboratory-confirmed infections who reported raw milk consumption (n = 530) was 25 times greater than the number of raw milk–associated outbreak cases (n = 21) among Minnesota residents. Furthermore, we estimated that up to 20,502 Minnesotans, or 17% of raw milk consumers, may have become ill with enteric pathogens during the study period after consuming raw milk. This finding suggests that outbreaks represent a small number of the illnesses associated with raw milk consumption and that the risk for illness associated with raw milk consumption is far greater than determined based on the occurrence of recognized outbreaks. Findings such as ours should be used to further educate potential raw milk consumers, as well as policy makers who might be asked by constituents to relax regulations regarding raw milk sales.
